# A Stratified Meta-Analysis of the Association between Exposure to Environmental Tobacco Smoke during Childhood and Adulthood and Urothelial Bladder Cancer Risk

**DOI:** 10.3390/ijerph15040569

**Published:** 2018-03-22

**Authors:** Frits H. M. van Osch, Sylvia H. J. Jochems, Anke Wesselius, Frederik J. van Schooten, Richard T. Bryan, Maurice P. Zeegers

**Affiliations:** 1 Department of Complex Genetics, Nutrition and Translational Research in Metabolism (School NUTRIM), Maastricht University, PO Box 616, 6200MD Maastricht, The Netherlands; s.jochems@maastrichtuniversity.nl (S.H.J.J.); anke.wesselius@maastrichtuniversity.nl (A.W.); m.zeegers@maastrichtuniversity.nl (M.P.Z.); 2 Institute of Cancer and Genomic Sciences, University of Birmingham, Birmingham B15 2TT, UK; r.t.bryan@bham.ac.uk; 3 Department of Pharmacology and Toxicology, NUTRIM School for Nutrition and Translational Research in Metabolism, Maastricht University, 6200MD Maastricht, The Netherlands; f.vanschooten@maastrichtuniversity.nl; 4 Department of Complex Genetics, Public Health and Primary Care (School CAPHRI), Maastricht University, 6200MD Maastricht, The Netherlands

**Keywords:** exposure to environmental tobacco smoke, bladder cancer, meta-analysis, lifetime exposure

## Abstract

*Background*: Active smoking is a major risk factor for urothelial bladder cancer (UBC). However, the evidence that exposure to environmental tobacco smoke (ETS) either in childhood or adult life is also associated with UBC risk is ambiguous. With this meta-analysis, we aim to summarise how exposure to ETS is associated with UBC risk. *Methods*: In total, 11 studies (3 cohort studies, 8 case-control studies) were included in this meta-analysis and summary odds ratios (SORs) for UBC risk were calculated for never smokers who were exposed to ETS during childhood at home, during adulthood at home, or during adulthood in a work environment compared to never smokers who were never exposed to ETS. Sensitivity analyses were conducted to test the robustness of findings. *Results*: Never smokers exposed to ETS during childhood (SOR = 1.04, 95% confidence interval (CI) = 0.82–1.26), during adulthood at work (SOR = 0.98, 95% CI = 0.78–1.18) or at home (SOR = 0.99, 95% CI = 0.83–1.15) were at a similar risk of UBC compared to never smokers who were never exposed to ETS. Results for males and females were similar. Also, when pooling all estimates during both childhood and adulthood, no effect was observed (SOR = 1.00, 95% CI = 0.89–1.10). *Conclusions*: Although measurement of exposure to ETS was imprecise, there does not seem to be an association between UBC risk and exposure to ETS during childhood or adulthood. However, the current body of evidence mostly overlooks the duration and intensity of exposure to ETS.

## 1. Introduction

Cigarette smoking is a major risk factor for urothelial bladder cancer (UBC) and accountable for a large proportion of UBC cases [1]. On average about 18% of adults smoke tobacco daily, with variation between countries worldwide [2]. Although the number of smokers has been decreasing over the past few decades, there are still a lot of individuals potentially exposed to environmental tobacco smoke (ETS). Although fewer cases of cancer can be attributed to exposure to ETS compared to active smoking, it is a type of exposure that can be prevented to a large extent.

A comprehensive Surgeon General’s report from 2006 on the health consequences of involuntary exposure to tobacco smoke in the United States described how homes and workplaces are the most common places for exposure to ETS, and that those with a relatively low income are more likely to be exposed to ETS [3]. Moreover, a retrospective analysis of 192 countries showed that exposure to ETS was responsible for approximately 1% of global mortality in 2004 [4]. Interestingly, in both large-scale reports, the association between exposure to ETS and UBC risk was not assessed, while associations with lung, breast, cervical and nasopharyngeal cancer are summarized [3]. Even though smoking is the largest preventable risk factor for UBC, the impact of exposure to ETS on UBC risk remains underreported and unclear compared to other smoking-related cancers.

A 2016 meta-analysis on exposure to ETS and the risks of developing cancers other than lung or breast revealed no significant association with UBC in cohort studies (OR = 0.99, 95% confidence interval (CI) = 0.75–1.31), case–control studies (OR = 1.17, 95% CI = 0.99–1.39) or all included studies (OR = 1.13, 95% CI = 0.98–1.30) [5]. However, in this analysis childhood exposure was not assessed specifically, and not all studies that were pooled indicated whether exposure to ETS was at home (e.g., from a spouse or cohabitant) or at work. Moreover, there are several reports that exposure to ETS is widespread in both childhood and adolescents in for example the U.S. [6] and in adults in Greece [7]. With our meta-analysis, we aim to provide and in-depth analysis of the effects of the exposure to ETS on UBC risk stratified by timing of exposure (childhood vs. adulthood exposure) and location of adulthood exposure to ETS (workplace or domestic exposure).

## 2. Methods

### 2.1. Literature Search

Several online databases (Medline and Embase) were used to search for epidemiologic studies on exposure to ETS and UBC incidence. Search strategies included search terms such as “urinary bladder neoplasms”, “incidence”, “risk”, “passive smoking”, or “exposure to environmental tobacco smoke”, and resulted in a total count of 110 articles after checking for duplicates. Additionally, cited articles in relevant reviews were checked to see whether no publications were missed. Articles were only included when they concerned human research on the association between ETS and the risk of UBC (primarily bladder cancer) and when risk estimates for UBC with 95% confidence intervals (CIs) were provided in tables or were potentially derivable from the text. 

### 2.2. Data Collection

All relevant papers published until December 2017 were assessed. In order to extract information on possible sources of heterogeneity and assess quality of included observational studies the Newcastle Ottawa scale [8] was applied to included publications by two authors (Frits H. M. van Osch and Sylvia H. J. Jochems). Data were either extracted directly from publications or ORs were estimated using manual calculation. When 95% CI’s had to be estimated an online tool was used [9]. If estimates for ETS exposure were only presented at different exposure levels the Excel spreadsheet described by Hamling et al. was used to obtain an overall risk estimate [10]. To establish the effect of ETS on UBC risk, all relevant data on risk estimates of UBC for exposure to ETS was collected by two independent researchers (i.e., Frits H. M. van Osch and Sylvia H. J. Jochems). These include estimates on childhood and adulthood separately as well as combined lifetime exposure estimates. For the stratified analysis, the data had to strictly state where (at home, by a spouse or other co-habitant or at work) and when (childhood or adulthood) exposure took place for inclusion. Furthermore, data on year of publication, geographic area (North America, Europe, Asia, Africa, South America), exposure to ETS assessment (interview or questionnaire) and case and control source (hospital, population or both) was extracted.

### 2.3. Statistical Analysis

A random effects meta-analysis was performed because some heterogeneity in true effect of exposure to ETS is to be expected between study populations who were exposed to different types of tobacco. Additionally, funnel plots investigating publication bias and the Egger’s test for small study effects were employed, as well as the I^2^ test for heterogeneity. All analyses were performed stratified for timing of exposure (childhood or adulthood) and for location of exposure (domestic or at work). Adjusted ORs that were pooled had to be at least adjusted for age when taking the questionnaire and gender. Sensitivity analyses were performed by pooling all different types of exposure to ETS in one analysis and by pooling only case-control studies. All analyses were performed using Stata statistical software (version 14; Stata Corp., College Station, TX, USA).

## 3. Results

### 3.1. Study Characteristics

Following full text evaluation, 14 articles initially met the inclusion criteria. None of the articles were excluded based on their quality assessment as all studies scored at least 7 out of 9 on the Newcastle-Ottawa scale. Of these 14 articles, two were excluded because it was not explicitly mentioned what the nature (timing and location) of exposure to ETS was [11,12]. Another study only showed results for urinary tract cancer and was therefore excluded [13]. The remaining 11 publications, containing data from 12 populations, were included in our final analysis (Table 1). Studies were mostly from Western countries, except for one study from China [14]. Three cohort studies [15,16,17] and eight case-control studies [14,18,19,20,21,22,23,24] were identified. Of these studies, the majority focussed on (urothelial) bladder cancer, although one study combined all urothelial cancers (bladder, uterus, renal pelvis or urethra) [15]. Furthermore, in the analysis domestic exposure to ETS data was pooled for any household members; studies which indicated spouses and other household members specifically were pooled to provide one risk estimate.

### 3.2. Pooled Results from Stratified Analysis

**Figure 1:**
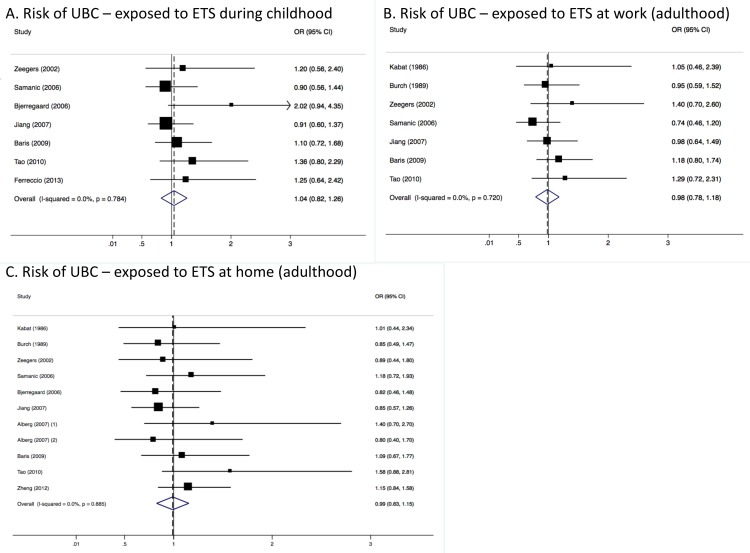
Meta-analysis results showing pooled risk estimates for urothelial bladder cancer (UBC) stratified by timing (childhood or adulthood) and location (work-related or domestic) of exposure to ETS for males and females combined.

Only seven studies reported estimates on childhood exposure to ETS. Pooling UBC risk estimates from these studies resulted in a summary odds ratio (SOR) of 1.04 (95% CI = 0.82–1.26) (Figure 1, panel A) for both genders combined. There were also seven studies that provided estimates of UBC risk (both genders combined) for never smokers exposed to ETS at work compared to those who were never exposed to ETS at work, which also showed no significant differences in UBC risk (SOR = 0.98, 95% CI = 0.78–1.18) (Figure 1, panel B). Ten of the included studies estimated UBC risk for those exposed to ETS at home because of living with a spouse or any other cohabitant. Pooling the results from these studies resulted in an SOR of 0.99 (95% CI = 0.83–1.15), also indicating no significant impact on UBC risk for those exposed to ETS (Figure 1, panel C).

### 3.3. Heterogeneity and Publication Bias

**Figure 2:**
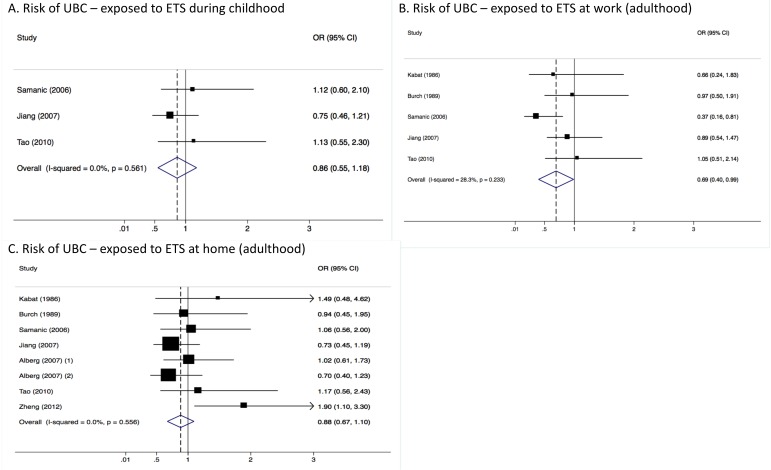
Meta-analysis results showing pooled risk estimates for urothelial bladder cancer (UBC) stratified by timing (childhood or adulthood) and location (work-related or domestic) of exposure to ETS for males only.

**Figure 3:**
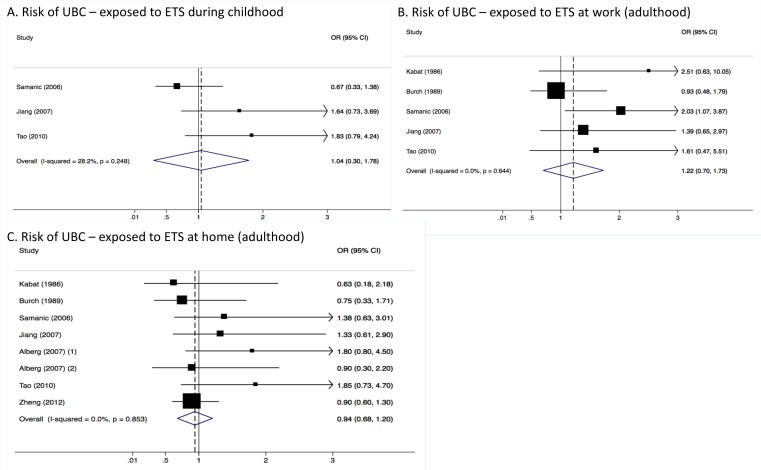
Meta-analysis results showing pooled risk estimates for urothelial bladder cancer (UBC) stratified by timing (childhood or adulthood) and location (work-related or domestic) of exposure to ETS for females only.

Heterogeneity between studies included in the analyses was very low, with an I^2^ of 0.0% (*p*-value > 0.05 in all three analyses) (Figure 1). Results were similar for males (Figure 2) and females (Figure 3). Although in men only, those exposed to workplace exposure to ETS seemed to have a lower risk of UBC (SOR = 0.69, 95% CI = 0.40–0.99). However, this was strongly driven by a low risk estimate obtained from one study (Figure 2). However, this further stratification of the pooled risk estimates also showed the uncertainty in the analysis, for example in the broad confidence intervals for UBC risk in those exposed to ETS during childhood in males (SOR = 0.86, 95% CI = 0.55–1.18) and females (SOR = 1.04, 95% CI = 0.30–1.78). Generally, the pooled estimates for those exposed to ETS at home (panel C in Figure 1, Figure 2 and Figure 3) were most stable (SOR_total_ = 0.99, SOR_males_ = 0.88 and SOR_females_ = 0.94).

Egger’s test for small-study effects indicated no publication bias by excluding estimates for smaller studies compared to larger studies in all three stratified analyses (*p* > 0.05 for all three analyses). Funnel plots also showed that all extracted risk estimates were within expected range of standard error of the pooled estimate based on their study’s sample size (Supplemental Figure S1). When excluding risk estimates from the three included cohort studies and thus analysing only case-control studies, the SOR remained the same as in the overall analysis (SOR_childhood_ = 1.01, 95% CI = 0.79–1.24, SOR_work_ = 0.98, 95% CI = 0.77–1.18 and SOR_home_ = 1.02, 95% CI = 0.83–1.21), as shown in Supplemental Figure S2. Furthermore, when pooling all estimates of UBC risk regardless of timing or location of exposure to ETS there also did not seem to be any significant impact of exposure to ETS on UBC risk (SOR = 1.00, 95% CI = 0.89–1.10) or any heterogeneity caused by pooling estimates of exposure at different times in life (I^2^ = 0%, *p* = 0.978).

## 4. Discussion

The results from this meta-analysis indicate no substantial effect of exposure to ETS during either childhood or adulthood on UBC risk in never smokers. 

Although the heterogeneity among the included studies was very low and statistical power sufficient, the pooled UBC risk estimates obtained on exposure to ETS are very likely influenced by recall bias (especially when estimating childhood exposure). It is often demonstrated that large, prospective studies that report results for exposure to ETS and lung cancer risk do not report corresponding results for UBC risk [5].

Moreover, detailed information on the nature of exposure is often lacking. Studies rarely assess for how many years never smokers were exposed to ETS and whether the active smoker smoked daily or only sporadically. Therefore, risk estimates in the included studies are certainly confounded by the length of exposure and the smoking behaviour of the active smoker(s) providing the exposure to the never smoking subject.

Some of the included studies on exposure to ETS and UBC risk (included in this meta-analysis) have attempted to assess lifetime exposure to ETS and correct for length of exposure. Tao et al. described a combined index estimating lifetime exposure to ETS where different scores were added up for each member of the household that smoked (based on the number of cigarettes they smoked) and the hours of exposure to workplace ETS. The highest exposure category (5 or higher on a scale from 0–10) compared to never exposed never smokers showed an OR of 3.00 (1.24–7.26) [14]. Jiang (2007) also estimated a cumulative index of ETS exposure (i.e., sum of childhood exposure and three levels of adulthood exposure (domestic, workplace and social). However, they showed no statistically significant associations with UBC risk either at intermediate exposure level (OR = 1.61, 95% CI = 0.81–3.08) or at highest exposure level (OR = 1.28, 95% CI = 0.61–2.48). Also, Baris et al. found no association between a combined index of adulthood exposure with UBC risk (high versus low exposure OR = 0.9, 95% CI = 0.4–2.0) [22]. Interestingly, adult men that did not smoke cigarettes or water pipe and were exposed to ETS both outside and at home gad a three times increased risk of UBC (95% CI = 1.5–5.9) compared to other nontobacco-using men who were not exposed to ETS in Egypt which as statistically significant [23]. Because of the different ways of calculating cumulative exposure (e.g., only in adulthood or different weights to scores concerning number of cigarettes smoked) these data could not be pooled. However, it is noticeable that all studies that estimated some form of cumulative exposure showed an increased UBC risk, often being statistically significant, and with higher risk estimates than any of the individual estimates pooled in Figure 1. More research is warranted in cumulative exposure to ETS to see whether those in the highest cumulative exposure categories during both childhood and adulthood might be at an increased risk of UBC.

Additionally, two studies that were not included in the meta-analysis because they did not indicate where exposure to ETS took place, reported ORs for UBC risk for both ever smokers and never smokers who were exposed to ETS combined [12,16]. Both studies show a markedly increased risk for those exposed to ETS regardless of their smoking status: in a Taiwanese case-control study the observed OR was 1.90 (95% CI = 1.42–2.55) and in a large European-wide cohort study the OR was 1.38 (95% CI = 1.00–1.90). Even though these estimates are likely confounded by current smoking status, it is striking that statistically significant risk estimates are observed in these studies which is rare considering the individual estimates pooled in this meta-analysis. It would be meaningful to also see more future research focussing on the effects of exposure to ETS in all subjects regardless of their smoking status.

Apart from the limited information that can be drawn from retrospectively gathered data, there are also possible confounders and effect modifiers that were not considered in the pooled studies. There is evidence of an interaction effect between arsenic methylation and exposure to ETS in determining UBC risk where only those with high total urinary arsenic level are at an increased risk of UBC [11,12], however both studies investigating this interaction were not included in the meta-analysis since they did not indicate what the timing and location of exposure to ETS was. Also, children of parents who smoke are more likely to start smoking themselves [25], possibly because nicotine receptors are also stimulated in the brain by second-hand smoke [26]. Therefore, the never smokers that were exposed to ETS during childhood but never started smoking themselves are probably a biased reference group which is less susceptible to nicotine addiction compared to those who started smoking after being exposed to ETS. However, more research is needed in ever smokers who were exposed to ETS during childhood to confirm this.

## 5. Conclusions

The current evidence suggests no substantial association between UBC risk and exposure to ETS either during childhood or adulthood. Nevertheless, the measurement of exposure to ETS was prone to bias since data was retrospectively collected in the included studies. More detailed information on duration and intensity of exposure to ETS is needed to answer the question whether there is also no association with UBC risk in high lifetime cumulative exposure to ETS categories or in ever smokers who were exposed to ETS.

## Figures and Tables

**Table 1:** Characteristics and type of extracted risk estimates of included case-control studies and cohort studies on exposure to environmental tobacco smoke (ETS) and urothelial by year of publication.

Reference	First Author	Year	Country	Never Smokers *	Study Design			Cigarette Smoking Assessment	Exposure to ETS		
					Cohort Study	Case-Control Study			Childhood Exposure	Adulthood Exposure	
						Case Source	Control Source			At Home	At Work
[18]	Kabat	1986	USA	644	-	Hospital	Hospital	Structured interview	-	Yes	Yes
[19]	Burch	1989	Canada	359	-	Hospital	Population	Structured interview	-	Yes	Yes
[15]	Zeegers	2002	The Netherlands	1233	Yes	-	-	Postal questionnaire	Yes	Yes	Yes
[20]	Samanic	2006	Spain	528	-	Hospital	Hospital	Postal questionnaire	Yes	Yes	Yes
[16]	Bjerregaard	2006	Europe	220,790	Yes	-	-	Postal questionnaire	Yes	Yes	-
[21]	Jiang	2007	USA	440	-	Population	Population	Structured interview	Yes	Yes	Yes
[17]	Alberg *	2007	USA	18,839/20,181	Yes	-	-	Postal questionnaire	-	Yes	-
[22]	Baris	2009	USA	547	-	Population	Population	Structured interview	Yes	Yes	-
[14]	Tao	2010	China	456	-	Population	Population	Structured interview	Yes	Yes	Yes
[23]	Zheng	2012	Egypt	678	-	Hospital	Population	Structured interview	-	Yes	Yes
[24]	Ferreccio	2013	Chile	307	-	Population	Population	Structured interview	Yes	-	-

* Publication describes two cohorts with identical data collection.

## Supplementary Materials

The following are available online at http://www.mdpi.com/1660-4601/15/4/569/s1, Figure S1: Funnel plots showing risk estimates from individual studies relative to the pooled OR for both analysis on childhood exposure to ETS and adulthood exposure to ETS (both males and females), Figure S2: Meta-analysis results showing pooled risk estimates for UBC stratified by timing (childhood or adulthood) and location (work-related or domestic) of exposure to ETS for males and females combined, only for case-control studies.
