# Supplementary material for: A Stratified Meta-Analysis of the Association between Exposure to Environmental Tobacco Smoke during Childhood and Adulthood and Urothelial Bladder Cancer Risk
Source: Int J Environ Res Public Health. 2018 Mar 22;15(4):569. doi: 10.3390/ijerph15040569 (PMC5923611; doi:10.3390/ijerph15040569)
Supplement: Supplementary file 1 [file ijerph-15-00569-s001.pdf]

Supplementary Materials: A Stratified Meta-Analysis of the Association between Exposure to Environmental Tobacco Smoke during Childhood and Adulthood and Urothelial Bladder Cancer Risk

Frits H. M. van Osch, Sylvia H. J. Jochems, Anke Wesselius, Frederik J. van Schooten, Richard T. Bryan and Maurice P. Zeegers

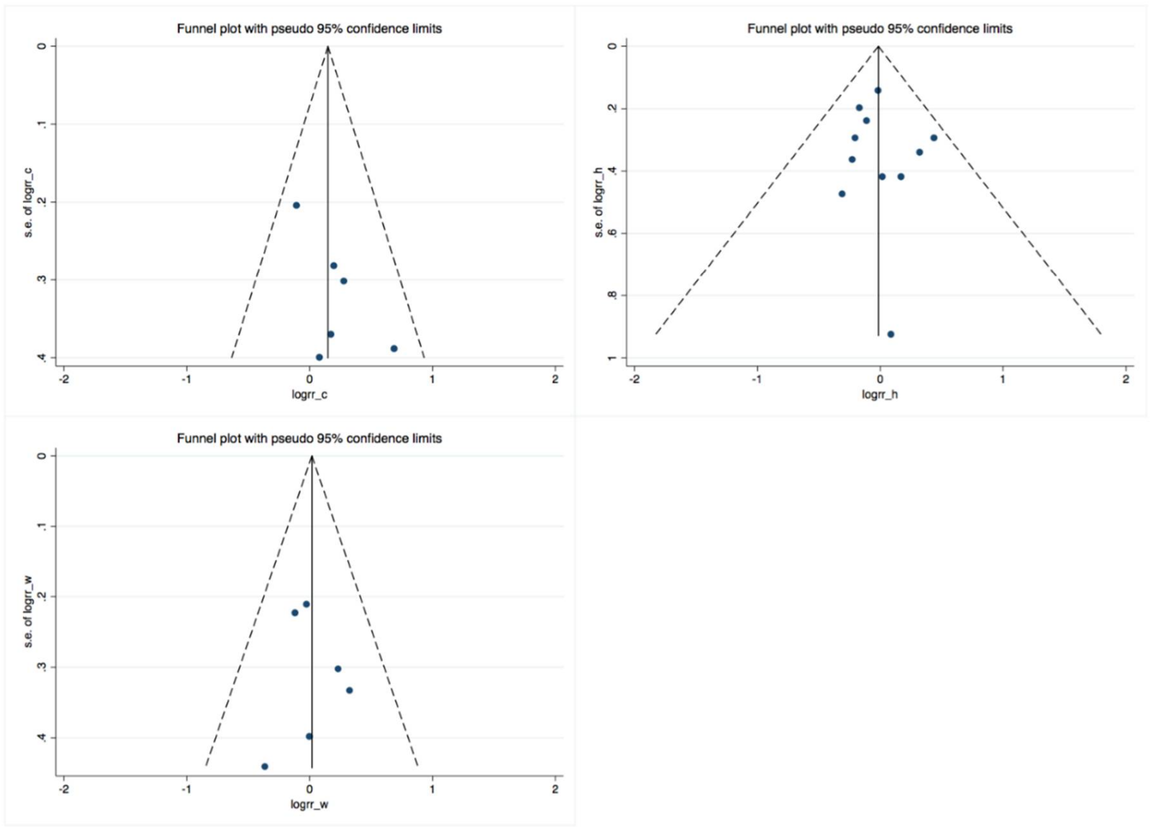

Figure S1. Funnel plots showing risk estimates from individual studies relative to the pooled OR for both analysis on childhood exposure to ETS and adulthood exposure to ETS (both males and females).

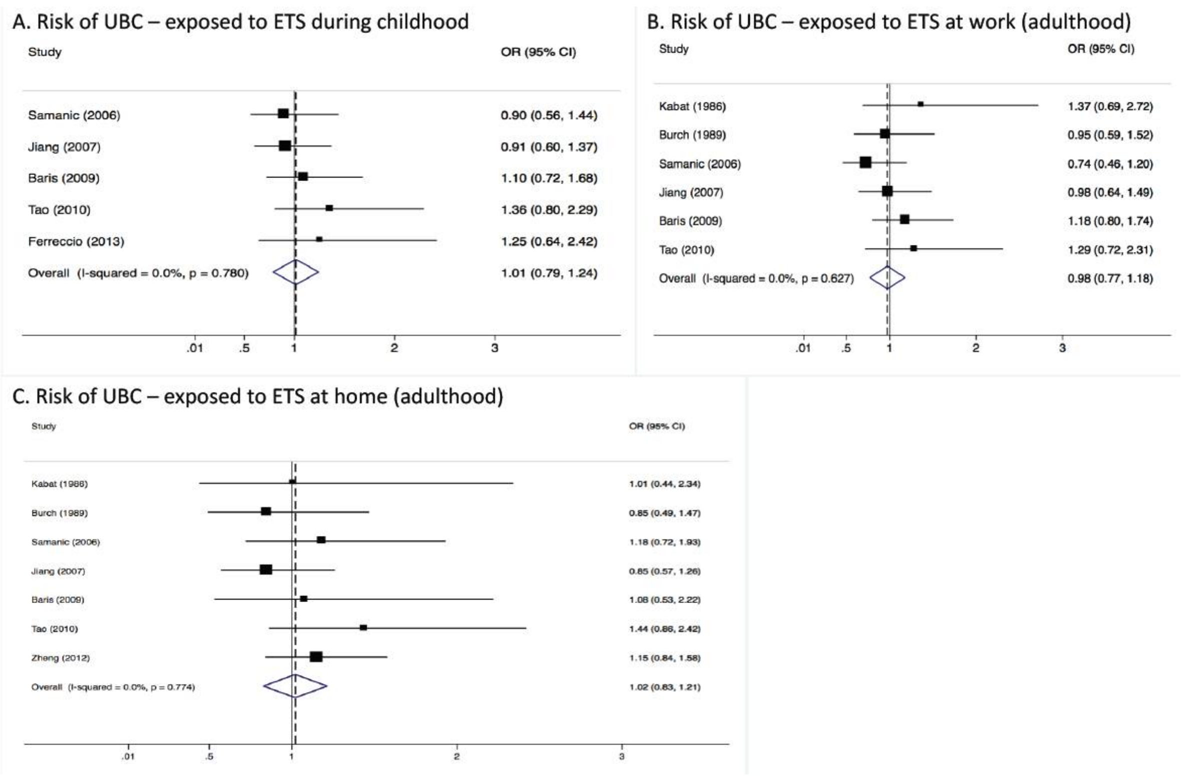

Figure S2. Meta-analysis results showing pooled risk estimates for UBC stratified by timing (childhood or adulthood) and location (work-related or domestic) of exposure to ETS for males and females combined, only for case-control studies.

© 2018 by the authors; licensee MDPI, Basel, Switzerland. This article is an open access article distributed under the terms and conditions of the Creative Commons by Attribution (CC-BY) license (http://creativecommons.org/licenses/by/4.0/).
